# Supplementary figures and images for: Identification of elite rice lines with better breeding values using genomic prediction and multi-trait genotype ideotype distance index (MGIDI) for grain yield under irrigation cropping system
Source: PLoS One. 2026 Feb 5;21(2):e0340188. doi: 10.1371/journal.pone.0340188 (PMC12875472; doi:10.1371/journal.pone.0340188)

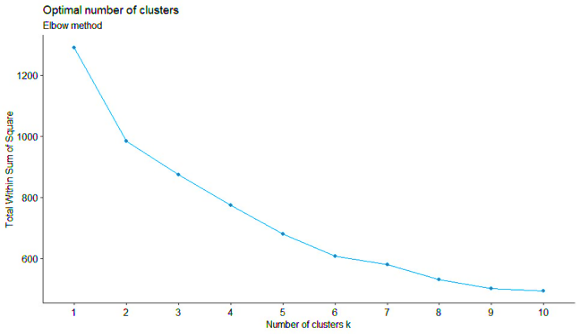


A) 2022WS (T.Aman)


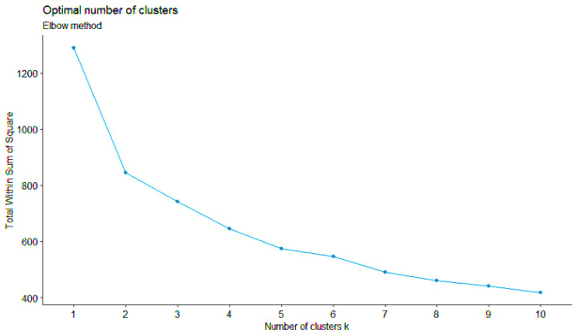


B) 2023DS (Boro)

S1 Figure. Elbow curve of K-means cluster.

Supplement: S1 Fig — (DOCX) [file pone.0340188.s001.docx]
