# Supplementary material for: Identification of elite rice lines with better breeding values using genomic prediction and multi-trait genotype ideotype distance index (MGIDI) for grain yield under irrigation cropping system
Source: PLoS One. 2026 Feb 5;21(2):e0340188. doi: 10.1371/journal.pone.0340188 (PMC12875472; doi:10.1371/journal.pone.0340188)
Supplement: S1 Table — (DOCX) [file pone.0340188.s002.docx]

S1 Table: List of Genotypes

| **Entry No.** | **Designation** | **Entry No.** | **Designation** | **Entry No.** | **Designation** |
| --- | --- | --- | --- | --- | --- |
| E001 | IR19A7804 | E039 | IR19A8100 | E077 | IR19A8925 |
| E002 | IR19A8814 | E040 | IR19A8104 | E078 | IR19A8964 |
| E003 | IR19A8832 | E041 | IR19A8115 | E079 | IR19A8982 |
| E004 | IR19A8834 | E042 | IR19A8117 | E080 | IR19A8989 |
| E005 | IR19A7339 | E043 | IR19A8121 | E081 | IR19A9000 |
| E006 | IR19A7340 | E044 | IR19A8124 | E082 | IR19A9067 |
| E007 | IR19A7347 | E045 | IR19A8127 | E083 | IR19A9089 |
| E008 | IR19A7352 | E046 | IR19A8129 | E084 | IR19A9101 |
| E009 | IR19A7361 | E047 | IR19A8130 | E085 | IR19A9113 |
| E010 | IR19A7362 | E048 | IR19A8167 | E086 | IR19A9129 |
| E011 | IR19A7386 | E049 | IR19A8172 | E087 | IR19A9165 |
| E012 | IR19A7401 | E050 | IR19A8175 | E088 | IR19A9166 |
| E013 | IR19A7406 | E051 | IR19A8251 | E089 | IR19A9247 |
| E014 | IR19A7453 | E052 | IR19A8258 | E090 | IR19A9250 |
| E015 | IR19A7501 | E053 | IR19A8260 | E091 | IR19A9256 |
| E016 | IR19A7510 | E054 | IR19A8275 | E092 | IR19A9257 |
| E017 | IR19A7511 | E055 | IR19A8278 | E093 | IR19A9261 |
| E018 | IR19A7523 | E056 | IR19A8298 | E094 | IR19A9207 |
| E019 | IR19A7531 | E057 | IR19A8305 | E095 | IR19A9211 |
| E020 | IR19A7541 | E058 | IR19A8318 | E096 | IR19A9212 |
| E021 | IR19A7558 | E059 | IR19A8333 | E097 | IR19A9279 |
| E022 | IR19A7560 | E060 | IR19A8334 | E098 | IR19A9287 |
| E023 | IR19A7601 | E061 | IR19A8583 | E099 | IR19A9294 |
| E024 | IR19A7620 | E062 | IR19A8520 | E100 | IR19A9298 |
| E025 | IR19A7624 | E063 | IR19A8523 | E101 | IR19A7803 |
| E026 | IR19A7633 | E064 | IR19A8535 | E102 | IR19A7808 |
| E027 | IR19A7664 | E065 | IR19A8604 | E103 | IR19A7810 |
| E028 | IR19A7683 | E066 | IR19A8605 | E104 | IR19A7812 |
| E029 | IR19A7710 | E067 | IR19A8612 | E105 | IR19A7813 |
| E030 | IR19A7729 | E068 | IR19A8614 | E106 | IR19A7815 |
| E031 | IR19A7733 | E069 | IR19A8617 | E107 | IR19A7816 |
| E032 | IR19A7742 | E070 | IR19A8624 | E108 | IR19A7817 |
| E033 | IR19A7951 | E071 | IR19A8653 | E109 | IR19A7818 |
| E034 | IR19A7957 | E072 | IR19A8664 | E110 | IR19A7828 |
| E035 | IR19A8047 | E073 | IR19A8784 | E111 | IR19A7830 |
| E036 | IR19A8052 | E074 | IR19A8842 | E112 | IR19A7831 |
| E037 | IR19A8054 | E075 | IR19A8850 | E113 | IR19A7832 |
| E038 | IR19A8066 | E076 | IR19A8864 | E114 | IR19A7833 |

| **Entry No.** | **Designation** | **Entry No.** | **Designation** | **Entry No.** | **Designation** | |
| --- | --- | --- | --- | --- | --- | --- |
| E115 | IR19A7834 | E154 | IR19A7588 | E193 | IR19A9069 | |
| E116 | IR19A7836 | E155 | IR19A7590 | E194 | IR19A9070 | |
| E117 | IR19A7839 | E156 | IR19A7591 | E195 | IR19A9072 | |
| E118 | IR19A7840 | E157 | IR19A7596 | E196 | IR19A9073 | |
| E119 | IR19A7843 | E158 | IR19A8202 | E197 | IR19A9074 | |
| E120 | IR19A7845 | E159 | IR19A8206 | E198 | IR19A9078 | |
| E121 | IR19A7408 | E160 | IR19A8211 | E199 | IR19A9080 | |
| E122 | IR19A7409 | E161 | IR19A8214 | E200 | IR19A9083 | |
| E123 | IR19A7410 | E162 | IR19A8215 | **Global check** | | |
| E124 | IR19A7411 | E163 | IR19A8226 | E201 | IRRI 104 | |
| E125 | IR19A7412 | E164 | IR19A8234 | E202 | IRRI 123 | |
| E126 | IR19A7414 | E165 | IR19A8237 | E203 | IRRI 154 | |
| E127 | IR19A7415 | E166 | IR19A8573 | E204 | IRRI 156 | |
| E128 | IR19A7419 | E167 | IR19A8576 | E205 | IRRI 168 | |
| E129 | IR19A7420 | E168 | IR19A8577 | E206 | IRRI 174 | |
| E130 | IR19A7427 | E169 | IR19A8578 | E207 | IR16A3838 | |
| E131 | IR19A7428 | E170 | IR19A8584 | E208 | IR16A3891 | |
| E132 | IR19A7429 | E171 | IR19A8588 | E209 | IR16A4085 | |
| E133 | IR19A7430 | E172 | IR19A8589 | E210 | IR16A4261 | |
| E134 | IR19A7432 | E173 | IR19A8590 | **Check for 2022WS** | | |
| E135 | IR19A7434 | E174 | IR19A8592 | E211 | | BRRI dhan75 |
| E136 | IR19A7437 | E175 | IR19A8593 | E212 | | BRRI dhan87 |
| E137 | IR19A7438 | E176 | IR19A8594 | E213 | | BRRI dhan49 |
| E138 | IR19A7440 | E177 | IR19A8596 | E214 | | Binadhan-11 |
| E139 | IR19A7550 | E178 | IR19A8597 | E215 | | Binadhan-17 |
| E140 | IR19A7553 | E179 | IR19A8598 | E216 | | Rabi Dhan-1 |
| E141 | IR19A7554 | E180 | IR19A8600 | **Check for 2023DS** | | |
| E142 | IR19A7555 | E181 | IR19A9045 | E211 | | BRRI dhan28 |
| E143 | IR19A7557 | E182 | IR19A9046 | E212 | | BRRI dhan29 |
| E144 | IR19A7566 | E183 | IR19A9047 | E213 | | BRRI dhan67 |
| E145 | IR19A7568 | E184 | IR19A9052 | E214 | | BRRI dhan88 |
| E146 | IR19A7570 | E185 | IR19A9053 | E215 | | BRRI dhan89 |
| E147 | IR19A7571 | E186 | IR19A9054 | E216 | | BRRI dhan92 |
| E148 | IR19A7575 | E187 | IR19A9058 |  |  |  |
| E149 | IR19A7578 | E188 | IR19A9059 |  |  |  |
| E150 | IR19A7580 | E189 | IR19A9061 |  |  |  |
| E151 | IR19A7581 | E190 | IR19A9062 |  |  |  |
| E152 | IR19A7583 | E191 | IR19A9063 |  |  |  |
| E153 | IR19A7586 | E192 | IR19A9068 |  |  |  |
