# Supplementary material for: Identification of elite rice lines with better breeding values using genomic prediction and multi-trait genotype ideotype distance index (MGIDI) for grain yield under irrigation cropping system
Source: PLoS One. 2026 Feb 5;21(2):e0340188. doi: 10.1371/journal.pone.0340188 (PMC12875472; doi:10.1371/journal.pone.0340188)
Supplement: S2 Table — (DOCX) [file pone.0340188.s003.docx]

S2 Table. Analysis of variance (ANOVA) of nine morphological traits & mean performance of the breeding lines in 2022WS and 2023DS

| Season | 2022WS | | | | 2023DS | | | |
| --- | --- | --- | --- | --- | --- | --- | --- | --- |
| Traits | Mean | Minimum | Maximum | F value | Mean | Minimum | Maximum | F value |
| DFF | 87.52 | 70.93 | 97.50 | *** | 119.54 | 110.52 | 131.58 | *** |
| DTM | 118.86 | 101.00 | 130.00 | *** | 151.04 | 143.00 | 160.00 | *** |
| PH | 117.60 | 84.00 | 146.00 | *** | 103.89 | 75.00 | 133.00 | *** |
| ETH | 8.93 | 6.00 | 14.00 | *** | 11.78 | 8.00 | 20.00 | *** |
| PL | 27.28 | 20.17 | 34.67 | *** | 24.41 | 19.67 | 31.00 | *** |
| SFT | 73.82 | 46.26 | 94.81 | *** | 77.27 | 51.10 | 95.69 | *** |
| LWR | 5.45 | 4.43 | 6.67 | *** | 5.19 | 4.46 | 6.19 | *** |
| TGW | 25.1 | 18.1 | 31.5 | *** | 23.6 | 18.0 | 35.0 | *** |
| YTH | 3.39 | 0.74 | 5.34 | *** | 6.04 | 2.50 | 8.67 | *** |
