# Supplementary material for: Identification of elite rice lines with better breeding values using genomic prediction and multi-trait genotype ideotype distance index (MGIDI) for grain yield under irrigation cropping system
Source: PLoS One. 2026 Feb 5;21(2):e0340188. doi: 10.1371/journal.pone.0340188 (PMC12875472; doi:10.1371/journal.pone.0340188)
Supplement: S3 Table — (DOCX) [file pone.0340188.s004.docx]

S3 Table. List of Genomic estimated breeding values (GEBV)

| **Designation** | **GEBV** | **Designation** | **GEBV** | **Designation** | **GEBV** |
| --- | --- | --- | --- | --- | --- |
| IR19A8054 | 0.68190403 | IR19A8520 | 0.11285451 | IR19A9298 | 0.02564203 |
| IR19A8052 | 0.64951309 | IR19A7406 | 0.1078852 | IR19A8167 | 0.02547616 |
| IR19A7501 | 0.64293523 | IR19A7566 | 0.09421151 | IR19A7414 | 0.02387444 |
| IR19A8047 | 0.63392122 | IR19A7558 | 0.09315273 | IR19A9061 | 0.02318382 |
| IR19A8066 | 0.62400483 | IR19A7568 | 0.08792508 | IR19A7729 | 0.02234815 |
| IR19A7531 | 0.59873685 | IR19A7401 | 0.08549145 | IR19A9247 | 0.02089218 |
| IR19A7523 | 0.5829385 | IR19A7555 | 0.08319102 | IR19A7664 | 0.01909363 |
| IR19A7541 | 0.55783956 | IR19A9059 | 0.08297147 | IR19A8334 | 0.0170632 |
| IR19A7510 | 0.53327839 | IR19A9069 | 0.08082301 | IR19A7828 | 0.01595389 |
| IR19A8278 | 0.34391584 | IR19A9047 | 0.08023207 | IR19A7430 | 0.01440995 |
| IR19A9207 | 0.33324462 | IR19A7553 | 0.07987685 | IR19A7581 | 0.01357702 |
| IR19A8298 | 0.3325217 | IR19A7557 | 0.07973503 | IR19A9045 | 0.01327767 |
| IR19A9212 | 0.32330795 | IR19A7560 | 0.07772194 | IR19A9089 | 0.01286425 |
| IR19A9287 | 0.30842506 | IR19A7511 | 0.0711469 | IR19A7833 | 0.01281862 |
| IR19A8214 | 0.30135862 | IR19A7583 | 0.07065993 | IR19A7742 | 0.01173288 |
| IR19A7415 | 0.26721656 | IR19A8104 | 0.06863645 | IR19A8206 | 0.01074613 |
| IR19A7432 | 0.23894202 | IR19A7340 | 0.0653894 | IR19A9166 | 0.01069257 |
| IR19A9070 | 0.23618225 | IR19A9067 | 0.06156529 | IR19A7840 | 0.01010678 |
| IR19A7429 | 0.23316944 | IR19A7586 | 0.06100626 | IR19A7831 | 0.00998712 |
| IR19A7408 | 0.22151958 | IR19A7575 | 0.0533156 | IR19A9078 | 0.00877471 |
| IR19A7411 | 0.21159182 | IR19A8127 | 0.04955199 | IR19A9074 | 0.0069912 |
| IR19A7550 | 0.20442846 | IR19A9261 | 0.0483006 | IR19A9294 | 0.00666164 |
| IR19A9054 | 0.19270626 | IR19A8202 | 0.0482855 | IR19A7591 | 0.00347934 |
| IR19A7410 | 0.19185997 | IR19A7588 | 0.04726154 | IR19A9257 | 0.00040786 |
| IR19A7362 | 0.18614882 | IR19A8117 | 0.04718203 | IR19A7832 | -0.0021938 |
| IR19A8275 | 0.18536498 | IR19A7590 | 0.04547826 | IR19A8318 | -0.0022469 |
| IR19A9062 | 0.18305411 | IR19A7440 | 0.04414914 | IR19A8226 | -0.0025585 |
| IR19A9083 | 0.17994655 | IR19A7834 | 0.04066285 | IR19A7818 | -0.0046303 |
| IR19A9073 | 0.17865659 | IR19A7453 | 0.04023247 | IR19A8989 | -0.0078383 |
| IR19A7571 | 0.16827852 | IR19A7580 | 0.03890415 | IR19A8784 | -0.0099976 |
| IR19A7409 | 0.16451331 | IR19A9250 | 0.03643925 | IR19A9101 | -0.0099989 |
| IR19A7339 | 0.16186238 | IR19A8523 | 0.0362039 | IR19A9129 | -0.0117365 |
| IR19A9080 | 0.15790018 | IR19A7578 | 0.03512976 | IR19A7815 | -0.0147618 |
| IR19A7427 | 0.15787656 | IR19A8333 | 0.03371116 | IR19A7813 | -0.015594 |
| IR19A8964 | 0.1559469 | IR19A9063 | 0.03361189 | IR19A8130 | -0.0166171 |
| IR19A9211 | 0.14883301 | IR19A7843 | 0.03359483 | IR19A8664 | -0.0175761 |
| IR19A7412 | 0.14584239 | IR19A7733 | 0.03237374 | IR19A7386 | -0.0206998 |
| IR19A7347 | 0.12075606 | IR19A9053 | 0.02875548 | IR19A8172 | -0.0238345 |

| **Designation** | **GEBV** | **Designation** | **GEBV** | **Designation** | **GEBV** |
| --- | --- | --- | --- | --- | --- |
| IR19A7438 | -0.026011 | IR19A8211 | -0.1488091 | IR19A8598 | -0.2904429 |
| IR19A9000 | -0.0272281 | IR19A7957 | -0.1499471 | IR19A8834 | -0.333192 |
| IR19A7812 | -0.0286829 | IR19A8175 | -0.1563519 | IR19A7804 | -0.3587473 |
| IR19A7596 | -0.0289834 | IR19A8115 | -0.1673871 | IR19A8624 | -0.4264475 |
| IR19A7830 | -0.0295367 | IR19A9256 | -0.1697347 | IR19A8604 | -0.4692586 |
| IR19A7361 | -0.0326271 | IR19A8215 | -0.1771708 | IR19A8617 | -0.4737451 |
| IR19A7839 | -0.033872 | IR19A9058 | -0.1795095 | IR19A8605 | -0.4782869 |
| IR19A8234 | -0.03692 | IR19A8121 | -0.1825077 | IR19A8612 | -0.5265657 |
| IR19A8129 | -0.0378183 | IR19A8577 | -0.1843236 | IR19A8614 | -0.5526967 |
| IR19A7570 | -0.038271 | IR19A8593 | -0.1846675 |  |  |
| IR19A7836 | -0.0400145 | IR19A7808 | -0.1889986 |  |  |
| IR19A7810 | -0.0404052 | IR19A8237 | -0.1914758 |  |  |
| IR19A8653 | -0.0433503 | IR19A8590 | -0.1977245 |  |  |
| IR19A9113 | -0.0457564 | IR19A8305 | -0.1980004 |  |  |
| IR19A7817 | -0.0460684 | IR19A7633 | -0.1989072 |  |  |
| IR19A7428 | -0.0479773 | IR19A8576 | -0.1990476 |  |  |
| IR19A7554 | -0.0480719 | IR19A8594 | -0.1990837 |  |  |
| IR19A7352 | -0.0486722 | IR19A7683 | -0.202335 |  |  |
| IR19A7816 | -0.049 | IR19A8842 | -0.2023437 |  |  |
| IR19A9165 | -0.0496411 | IR19A9046 | -0.2031482 |  |  |
| IR19A7419 | -0.0507403 | IR19A8814 | -0.2050654 |  |  |
| IR19A7420 | -0.0513194 | IR19A8596 | -0.2133595 |  |  |
| IR19A9068 | -0.0519939 | IR19A8592 | -0.217189 |  |  |
| IR19A7624 | -0.0546763 | IR19A8124 | -0.2181351 |  |  |
| IR19A9072 | -0.0567632 | IR19A8251 | -0.2191311 |  |  |
| IR19A8925 | -0.0604344 | IR19A8589 | -0.2248338 |  |  |
| IR19A7434 | -0.0697026 | IR19A8258 | -0.226254 |  |  |
| IR19A8832 | -0.078482 | IR19A8578 | -0.2326664 |  |  |
| IR19A9279 | -0.0801586 | IR19A8573 | -0.2338098 |  |  |
| IR19A7601 | -0.0854032 | IR19A8584 | -0.2453173 |  |  |
| IR19A8982 | -0.0942297 | IR19A7710 | -0.2464932 |  |  |
| IR19A8850 | -0.1027779 | IR19A7951 | -0.2470713 |  |  |
| IR19A9052 | -0.1041114 | IR19A8260 | -0.2498206 |  |  |
| IR19A7437 | -0.1109134 | IR19A8597 | -0.2550757 |  |  |
| IR19A8535 | -0.1196021 | IR19A8583 | -0.2587525 |  |  |
| IR19A8100 | -0.1249317 | IR19A8864 | -0.2634957 |  |  |
| IR19A7620 | -0.126025 | IR19A8588 | -0.2743348 |  |  |
| IR19A7845 | -0.1361115 | IR19A7803 | -0.2788868 |  |  |
